# MBE Transitions to the Open Access Publication Model in 2021

**DOI:** 10.1093/molbev/msaa269

**Published:** 2020-12-10

**Authors:** Sudhir Kumar, Heather Rowe

**Affiliations:** 1 Institute for Genomics and Evolutionary Medicine, Temple University, Philadelphia, PA; 2 Department of Biology, Temple University, Philadelphia, PA

## Abstract

From the first day of 2021, all manuscripts published in the journal *Molecular Biology and Evolution* (MBE) will be freely accessible online without a subscription. This exciting change will make all the MBE content available to all readers immediately upon publication.

MBE’s Open Access (OA) policy, developed in partnership with the Oxford University Press (OUP), will be comprehensive. MBE allows authors to post the original, unrefereed version (the preprint) of their manuscript before formal editorial acceptance on any repository or website. All articles are automatically deposited in PubMed Central upon issue publication. Article text, associated metadata, and supporting information are made electronically accessible by OUP, a nonprofit publisher with a long history of supporting academic publishing. MBE authors already hold the copyright to their published materials with no restrictions. Authors may choose a publication license that is most compatible with their institutions’ policies and funding bodies. MBE’s OA policy makes it possible for authors to comply with varied OA initiatives, emerging demands of funding bodies, and national directives.

The transition to OA ends the 37-year tradition of MBE as a print journal that started in December 1983 (fig. 1). MBE adopted a hybrid publishing model in 2006. Authors have been able to choose OA by paying an article processing charge (APC). MBE’s freely accessible content has increased over time (fig. 2). Besides the substantial fraction of research manuscripts published under our open access option, MBE has made invited reviews, perspectives, protocols, and editorials freely available for all readers. All other MBE content has become freely available under a 12-month delay model for those who did not access articles through an institutional or individual subscription.

**Fig. 1:**
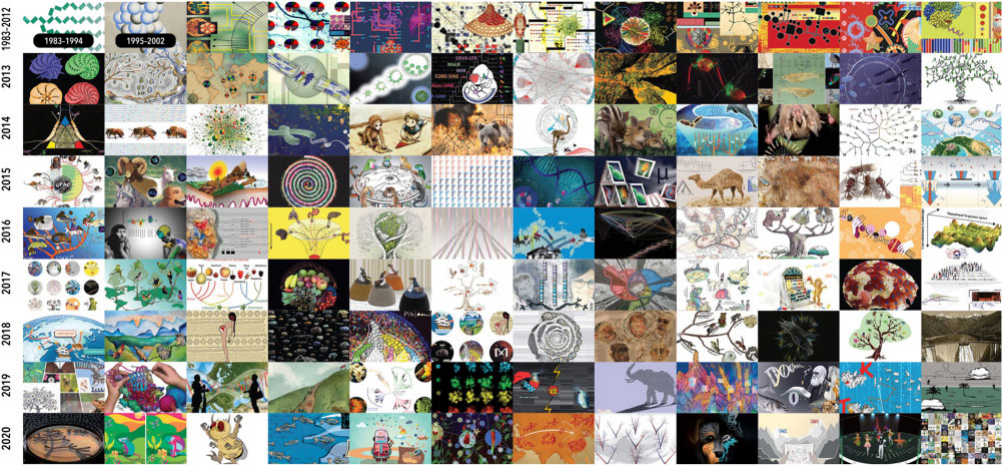
A collection of MBE covers graphics over the years. The first MBE issue was printed in December 1983. A new cover design was released in 1995 that was used until 2002. From 2003, a fresh image adorned MBE cover annually until 2012. From 2013, MBE published a new cover image every month, selected from the publishing author’s submissions.

**Fig. 2:**
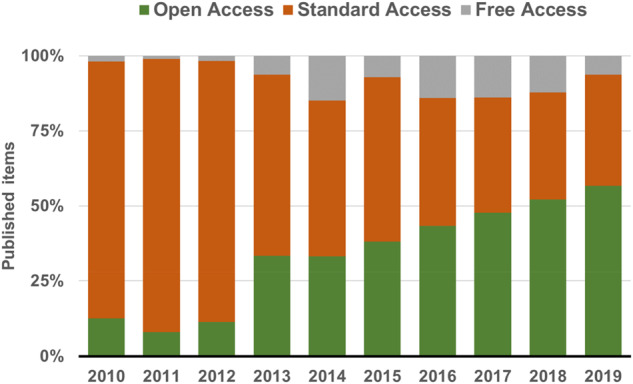
Increase in MBE’s Open Access and Free Access content over the last 10 years.

In many ways, MBE’s adoption of the OA model in 2021 is a natural next step in our journal’s evolution. The arguments in favor of OA publishing are compelling, as evidenced by the broad support for this publishing model in the scientific community. Indeed, scientific advances should be shared and discussed widely without financial barriers. The *Society for Molecular Biology and Evolution* (SMBE), which owns MBE, has a long history supporting OA publishing. In 2010, SMBE introduced *Genome Biology and Evolution* (GBE), an online-only journal published under an OA model. Yet the OA movement has not gained unanimous support, primarily because authors, institutions, and funding bodies must directly bear the cost of making their research widely accessible.

SMBE feels that the transition to OA for the journal will increase, not decrease, the access, participation, and impact of science shared through MBE with the global scientific community. At the same time, SMBE firmly believes that the publication of rigorous and high-impact research should not be dependent on researchers’ ability to pay APCs. Consequently, MBE enacted a policy of waiving page charges for the first ten printed pages of an article in 2016 to reduce the cost of traditional publication for authors in need. With MBE’s transition to full OA, relief from publication costs will now be made available through a generous policy of need-based APC waivers and discounts. The new system of waivers and discounts is entirely independent of the editorial process. It ensures that the ability to pay APC does not impact evaluating a manuscript’s suitability for publication in MBE. An independent committee will receive, evaluate, and approve requests for a waived or reduced fee for scientists working in developing countries and other scientists on a case-by-case basis (see https://academic.oup.com/mbe/pages/Open_Access).

Importantly, MBE APCs are among the least expensive compared with its peer journals. SMBE members receive a discount when publishing in MBE. MBE APCs defray the cost of publishing and providing need-based fee waivers. Any income beyond direct publication costs supports scientific knowledge and discourse supported by SMBE through society-sponsored activities. These activities include facilitating communication and interaction among its members, supporting outreach to the broader community, nurturing young scientists’ careers, enhancing diversity among our ranks of researchers, and celebrating research achievements. Specifically, SMBE spends proceeds from its journals to support annual meetings and smaller satellite meetings and workshops. SMBE supports conference registrations and travel for hundreds of students, young scientists, and established investigators every year. SMBE also confers honors on students and researchers studying principles, patterns, and applications of evolutionary biology.

It is the outstanding commitment of volunteer scientific editors and SMBE council members that enables SMBE to have resources to return to the community. These researchers donate their time, effort, and expertise, which is converted into support for SMBE’s community missions. In many OA journals, higher fees are charged without much broader benefit. SMBE and MBE are proud to provide a high impact, relatively inexpensive product that supports authors and the community at the same time. MBE is committed to working with our authors and readers to ensure publication of high-quality content in a financially sustainable manner for our Society. We aim to publish in the most inclusive way possible and welcome feedback from our community.

